# Examining the Unanswered Questions in TSW: A Case Series of 16 Patients and Review of the Literature

**DOI:** 10.3390/jcm15010361

**Published:** 2026-01-03

**Authors:** Max Y. Lu, Anna Erickson, Aditi Vijendra, Grace Ratley, Ashleigh A. Sun, Ian A. Myles, Nadia Shobnam

**Affiliations:** 1 Yale School of Medicine, New Haven, CT 06510, USA; 2 Epithelial Therapeutics Unit, National Institute of Allergy and Infectious Disease, National Institutes of Health, Bethesda, MD 20892, USA; 3 Integrative Metabolomics Unit, Institute of Environmental Medicine, Karolinska Institute, 171 77 Stockholm, Sweden; 4 Department of Internal Medicine, Hackensack Meridian School of Medicine, Nutley, NJ 07110, USA

**Keywords:** atopic dermatitis, corticosteroid, dermatology, topical, withdrawal

## Abstract

**Background/Objectives:** Topical steroid withdrawal syndrome is an underrecognized (and at times controversial) diagnosis, predominantly seen in individuals with a history of prolonged medium- to high-potency steroid use with sudden cessation. We aim to present topical steroid withdrawal clinical cases along with a narrative review of the literature to better characterize this understudied phenomenon. **Methods:** A total of 16 patients with a history of topical steroid withdrawal were enrolled in an IRB-approved clinical trial (NCT04864886). Participants underwent clinical assessments at the National Institutes of Health, including a history and physical examination, photography, genome sequencing, and comprehensive blood work. A follow-up survey assessed symptom activity and functional impact. **Results:** All patients reported severe itch, heat and photosensitivity, erythema, skin dryness, and pain. A total of 11 patients exhibited elevated IgE levels, 9 patients noted metallic-smelling skin, and 4 had peripheral blood eosinophilia. Symptomatic relief was observed with dupilumab, berberine, naltrexone, and various home remedies including topical ointments, vitamins, and probiotics, though effectiveness varied and often required trial and error. At follow-up, most respondents reported partial but ongoing symptoms, with several describing residual itch and intermittent interference with daily activities. Some participants continued therapeutic interventions, such as berberine, over two years after their initial evaluation. **Conclusions:** Our findings report improvement in patient symptoms such as itch and detail emerging management strategies that have not been discussed before. Improved recognition, physician consensus, and systemic evaluation of therapeutic options are needed to guide care and enhance quality of life for affected patients.

## 1. Introduction

Topical steroids are one of the most commonly prescribed treatments for inflammatory and pruritic dermatoses [1]. In recent decades and especially in recent years, there are increasing reports of adverse reactions following cessation of topical corticosteroids presenting with similar signs and symptoms, which have commonly been termed topical steroid withdrawal (TSW), otherwise referred to as red skin syndrome or topical steroid addiction [2]. Though first described in 1969, acceptance of this condition remains diffuse and contributes to the lack of formal diagnostic criteria [2]. Although a majority of dermatologists endorse TSW as distinct from atopic dermatitis (AD), surveys of British and Dutch healthcare professionals reveal over 15% still doubt TSW’s existence as a distinct entity [3,4,5]. Another survey of 70 Australian dermatologists found 59% did not consider TSW to be an entity [6], suggesting global variance in diagnosis.

Limited acceptance of TSW may stem from similarity of the signs and symptoms to many of the underlying dermatoses for which patients begin and subsequently cease topical steroid use, such as atopic or allergic contact dermatitis [2]. Similarly to other dermatoses, TSW often presents with erythema, papules, pruritus, burning or stinging, scaling, pustules, telangiectasias, edema, and dryness and flaking [7,8,9].

However, research has uncovered certain signs characteristic of the disease including the following: red skin, often spreading beyond areas where the topical corticosteroids were applied; red sleeves (redness extending from the shoulders and typically stopping at the margin of the dorsal and palmar (or solar) sides [10,11]); the headlight sign (facial erythema sparing the nose [10,12,13]); and elephant skin [10,12,13] (thickened, loose skin with reduced elasticity, often in extensor surfaces [10,13], which is distinct from lichenification of atopic dermatitis, which typically presents as leathery skin on flexor surfaces) [14,15]. Patients also often note hair loss or neuropathic pain, termed as “zingers” [13].

Still, given the lack of formal diagnostic criteria that complicates case identification, the characterization of TSW remains poor. Additional research is needed to further aid in TSW’s diagnosis, awareness, and management. In this article, we present a case series of 16 TSW patients, further adding to the body of literature on the condition’s presentation, emerging management strategies, and psychosocial impacts, including aspects previously not discussed. We also conducted a narrative synthesis of the literature to characterize the existing state of evidence surrounding TSW and to highlight gaps in knowledge.

## 2. Materials and Methods

Patients were recruited in collaboration with the International Topical Steroid Awareness Network and on clinical protocol NCT04864886, which was approved by the institutional review board of the National Institutes of Health. Written, informed consent for use of the images presented was provided by the participants.

Participants were eligible for inclusion if they had documented symptoms consistent with previous TSW case reports described in Shobnam et al. [13], were between 18 and 75 years of age, were willing to allow storage of blood, biopsy tissue, bacterial, and fungal cultures and other collected samples for future research, and were able to provide informed consent. Participants were excluded if they were currently or had recently (within the past three months) used anticoagulant or antiplatelet therapy other than aspirin or nonsteroidal anti-inflammatory drugs, had used immunomodulatory agents such as chemotherapy or steroids (unless approved by the principal investigator), had a history of keloid formation, were pregnant, lactating, or breastfeeding, or had any condition that, in the judgment of the investigator, would preclude safe participation in the study.

Patients were assessed at the National Institutes of Health Clinical Center (Bethesda, MD) and underwent a complete patient history and physical exam. Photograms were taken and some patients underwent genome sequencing and/or blood work with IgE levels and complete blood counts as previously described. Many patients elected to trial off-label therapies described in Table S1. Patients’ symptoms were reassessed at 5–8 months and again at 6–9 months after the initial presentation via telehealth or in-person follow-up. A cross-sectional follow-up survey (Google Forms) (Table S2) was distributed among the cohort via email in September 2025. The survey focused on current symptom activity and management approaches to characterize longer-term outcomes following initial assessment.

## 3. Results

### 3.1. Case Study

To illustrate the typical progression of TSW, we highlight Patient 1, a 27-year-old woman with no history of AD. At age 23, she noticed a rash spreading from her neck to her back and shoulders suspected to be an antibiotic drug allergy for which she was prescribed topical corticosteroids. She used this steroid for two months but did not recall the name nor potency. After the rash worsened, she was prescribed betamethasone, which she used on and off for a total steroid exposure time of 26 months. She also took a two-week oral course of prednisone with symptom improvement but subsequently experienced a worsening flare of her rash upon discontinuation. She was treated with tacrolimus for three months prior to self-discontinuation for suspected TSW. Initially, upon steroid cessation, her skin became erythematous, and like many other study participants, she experienced heat sensitivity, burning, severe pruritus, pain, and an oozing skin rash. She reported her skin had a “wet, metallic smell, almost like a wet coin.” Itch and pain were reported as her most debilitating symptoms with resultant insomnia. At the time of her NIH evaluation, she had been steroid-free for 19 months.

On initial evaluation, her lab work showed an elevated IgE level (>10,000). She was not on any daily oral medication or topical therapy at the time of the evaluation and only used an emollient (brand name Egyptian Magic) as well as a probiotic (brand name Probiotic 10). After initial evaluation, she was seen back in our clinic four months later. She was well for several months after her last visit but noticed a worsening rash correlating with increased stress. She did not report any other health-related problems and did not start new medications for the rash. During this visit she volunteered to begin taking an over-the-counter supplement, berberine, that may benefit TSW patients based on metabolomics and transcriptomics data from the initial pilot study [13]. Upon two months of berberine usage orally at 500 mg/d for one week, escalating to 1000 mg/d, the patient noticed her skin to be smoother and less flaky as well as an improved mood.

After three months on berberine, the patient saw skin improvement but still struggled with sleep. She was started on Doxepin (25 mg/night) to aid in sleep and itch while continuing with 1000 mg/d on berberine. It was suggested she start taking berberine baths every other day and applying Egyptian Magic to the skin. After two months of these baths, her skin appeared smoother, softer, and the redness decreased. These one-year results are parallel to a downward-trending absolute eosinophil count.

### 3.2. Cohort Characteristics

A total of 16 patients met the study’s inclusion criteria. Table 1 describes their age, gender, race, underlying condition, total steroid exposure, and time since discontinuation at the point of initial evaluation. Of the 16 patients, 14 had a prior history of AD. Of note is that the cohort was primarily women who identified as white. Mean time since steroid exposure at the time of initial evaluation was 17.2 years (median: 20 years; range: 2 years, 2 months–30 years). The mean time since discontinuation of steroids was 3.9 years (median: 2.4 years; range: 4 months–11 years). Notably, there was no history of inappropriate steroid usage as defined by use of high-potency steroids on the face or genitals [8] (Supplemental File S1).

### 3.3. Clinical Presentation and Symptoms

**Figure 1:**
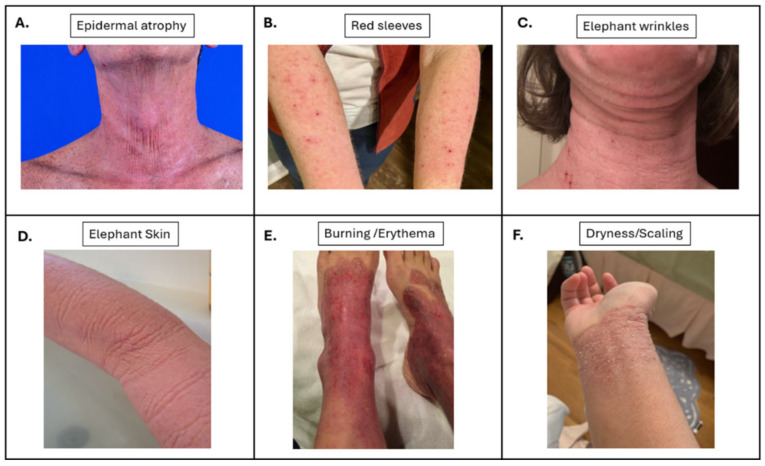
Representative images of TSW-associated symptoms and reported frequency of symptoms. (**A**). Epidermal atrophy, seen in Case 6, (**B**). “Red sleeves” seen in Case 3, (**C**). Elephant wrinkles, seen in Case 11, (**D**). Elephant skin, seen in Case 2, (**E**). Burning and erythema seen in Case 11, and (**F**). Dryness/scaling seen in Case 13.

Every patient experienced severe itch, heat/photosensitivity, erythema, skin dryness, and pain with the culmination feeling like a chemical burn (Figure 1). Onset of symptoms in patients ranged from within three days of stopping to an escalation over several months.

A total of 15 patients experienced swelling/edema and elephant skin. Additionally, 14 patients experienced nerve symptoms (zingers) and thermodysregulation. Of note is that 12 patients also described noting an unusual smell to their skin, with 9 patients characterizing this smell as metallic in nature. Some patients also described a metallic taste along with the smell (Table 2). Symptoms such as joint pain and insomnia were less frequent, but still present in 7 patients.

### 3.4. Treatment Approaches

Given the lack of established therapies for TSW, many patients in our study turned to off-label treatments. Six patients in our study had used dupilumab, with varying degrees of symptom relief. At the time of initial evaluation, two patients were actively using it with benefit, while two others had discontinued treatment after experiencing relief but developing side effects of conjunctivitis and facial vasculitis (Supplemental File S1). Another patient managed symptoms with the immunosuppressant cyclosporine, though with only minimal improvement. One patient trialed the JAK inhibitor upadacitinib for five months, achieving relief for just one month before the medication lost effectiveness, and another patient trialed the topical JAK inhibitor ruxolitinib for 8 weeks without substantial relief. Finally, one patient reported symptom relief with nighttime naltrexone.

To seek symptom relief, many patients also turned to over-the-counter and home remedies ranging from topical emollients to supplements such as vitamins, fish oils, or probiotics. Others also trialed naturopathic or homeopathic remedies. Patients often trialed extensive lists of dozens of products over the course of years, reporting subjective relief from some of these treatments, but often adverse effects from others, including burning, erythema, or worsening pruritis (Table S1).

In our case series of 16 patients, of the 13 who responded to telehealth or in-person follow-up, improvements were seen in 9 individuals after six months and 2 additional individuals by nine months. Improvements included a decrease in GI reflux, smoother skin, and less erythema, using a combination of ice cooling packs, berberine baths, and oral berberine. One patient still experienced significant symptoms despite two years of steroid avoidance and two patients lacked sufficiently detailed follow-up data to assess clinical progress.

### 3.5. Follow-Up Survey

Of the 16 patients in the cohort, 8 responded to the follow-up survey administered in September 2025, approximately 27 months since their initial NIH evaluation. During initial evaluation, patients were counseled on potential therapies including berberine and other treatments, several of which were trialed by participants (Table S1) [13]. Among the eight follow-up respondents, seven had trialed berberine while one had not. At the time of follow-up, two patients continued active use of berberine and another had last used it two years post-initial evaluation (Table S2). At follow-up, all participants continued to experience itch with it rated moderate or severe in half of respondents (Table 3). Half or more patients also reported impairments across domains including sleep, leisure/social activities, and household tasks/errands.

### 3.6. Laboratory Findings

**Figure 2:**
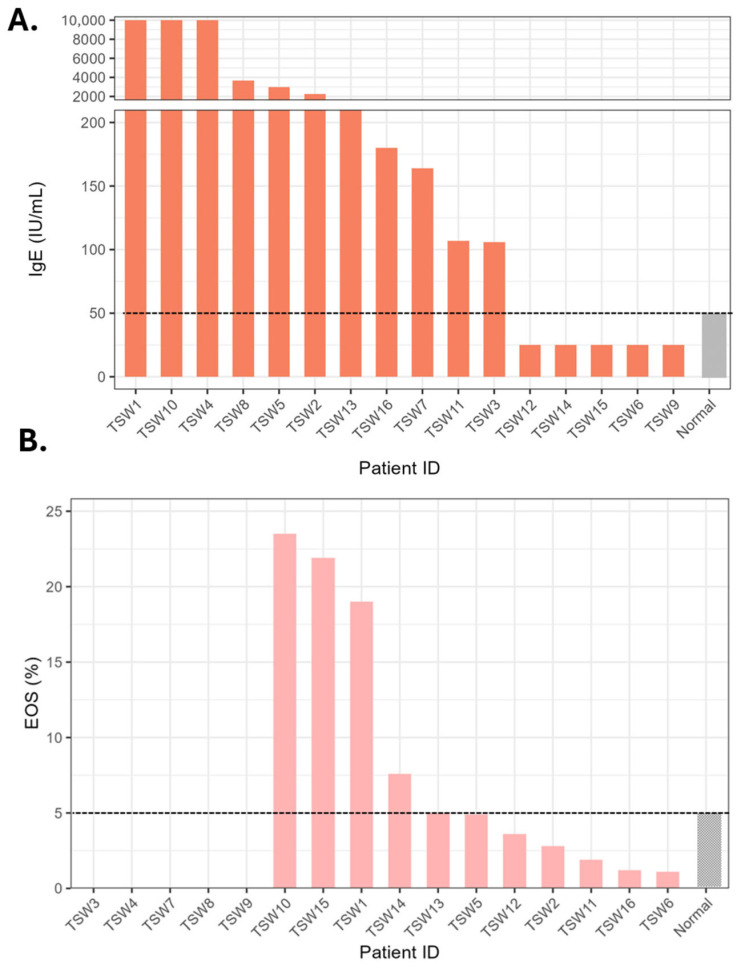
Peripheral blood eosinophil percentage (**A**) and serum IgE levels (**B**) across patients with topical steroid withdrawal. (**A**). Eosinophil percentages (EOS, %) are shown for each patient (TSW1-16). The gray bar indicates the normal reference range (0.7–5.8%), with several patients having levels over 10%. (**B**). Serum IgE (IU/mL) levels are displayed for the same patients. The gray bar indicates the normal reference threshold (≤50 IU/mL). A split-axis format is used to visualize both low-range values and elevated values. Measurements in the clinical laboratory used did not titrate beyond 10,000 IU/mL.

In addition to reporting symptomology, we also evaluated inflammatory profiles (Figure 2). Compared with a healthy reference range, 11 of 16 patients exhibited elevated IgE levels [16]. Although not all patients had complete blood counts, 4 of the 11 with eosinophil data demonstrated elevated peripheral eosinophil counts (absolute counts are found in Table S3) [17,18]. Two patients also experienced new-onset hypersensitivities to foods such as fish, pistachio, cashew, shrimp, and tomatoes, as well as sensitivity to sunscreen (Supplemental File S1, Cases 2 and 8). Both patients exhibiting these hypersensitivities had elevated IgE levels (TSW2 and TSW8).

## 4. Discussion

TSW is an increasingly recognized, though still poorly defined, condition that occurs after discontinuation of prolonged corticosteroid therapy. In our case series, many patients presented with common and previously characterized signs and symptoms of TSW including burning, heat sensitivity, pruritus, hot flashes, erythema, papules, pustules, swelling/edema, dryness, scaling, skin shedding, and neuropathic pain [7,8,9]. These symptoms overlap substantially with other dermatoses—most notably AD, which was present in 14 of the 16 patients in our series. Such overlap complicates evaluation and contributes to the lack of acceptance of TSW among many clinicians [2,3,4,5]. TSW may also resemble allergic contact dermatitis, thus patch testing is recommended to rule out this diagnosis [19]. However, this sometimes remains impractical given the large areas of red skin in TSW patients [12,20,21]. Even still, the use of patch testing may not entirely preclude a diagnosis of TSW, as it has been suggested that TSW may predispose patients to developing contact dermatitis, or exacerbate prior sensitivities [21,22]. In our cohort, no consistent pattern of ingredients could be identified, making undiagnosed contact dermatitis less likely, but not impossible. Furthermore, while worsening after TCS application may represent allergy to TCS or an excipient, our cohort avoided TCS for months to years prior to presentation in our clinic; the protracted time course after discontinuation thus precludes such a diagnosis.

However, certain features are more characteristic of TSW, including elephant skin (loose, thickened skin with reduced elasticity, often in extensor surfaces) [10,12,13] and red sleeves (redness extending from the shoulders and typically stopping at the margin of the dorsal and palmar (or solar) sides) [10,11]. Additionally, the clinical and morphological features are often different from the patient’s primary dermatoses and may extend beyond the region where topical steroids were originally applied [2,13]. Notably, in our case series, 9/16 of our patients also reported a metallic taste/metallic smelling skin, a symptom uncharacteristic of other dermatoses. Similarly, the neurologic symptoms of thermodysregulation, burning, and neuropathic pain (zingers) are not features of standard dermatoses. While no pathognomonic clinical signs for TSW have yet been identified, a recent analysis revealed that the presence of ≥1 major (burning, flushing, or thermodysregulation) and ≥3 minor criteria (bone deep itch, profuse peeling, red sleeves, loose skin, hair loss, zingers, lymph node swelling, or eye dryness) yielded >90% sensitivity for differentiating TSW from AD [13]. Ahuja and Lio (2025) have proposed criteria for diagnosing TSW as a diagnosis of exclusion [23]; however, in the absence of formalized criteria, both clinical care and research efforts remain challenging. It is important to consider the constellation of symptoms when considering TSW diagnosis. Each symptom may indeed be found in other disorders, but the overall picture presented in our cohort and the literature present an identifiable pattern.

One survey of dermatologists found 52.3% did not feel confident in diagnosing TSW [4]. This uncertainty surrounding diagnosis not only complicates clinical care but also poses a challenge for research, making it difficult to identify cases consistently, compare findings across studies, and establish reliable epidemiological data.

Beyond its dermatological manifestations, TSW imposes a significant psychosocial toll. Amongst our 16 patients, 7 experienced insomnia. Indeed, TSW is often associated with sleep as well as mood disturbances [7], including anxiety, depression, and suicidal thoughts [19]. Accordingly, many patients seek psychological support [8]. One study that included Dermatology Life Quality Index (DLQI) scoring for two patients found that TSW had “very large” effects on quality of life [19]. Upon follow-up in our study, 3 of 8 (37.5%) patients reported at least occasional effects of itch on leisure or social activities, household tasks, and work or school performance.

The etiology of TSW has yet to be elucidated, though it is typically associated with extended use of moderate- to high-potency corticosteroids, generally over six months or even a year and often on the face [7]. However, cases of TSW following low-potency corticosteroid use have been reported [8,9,24,25]. In our case series, the mean time of steroid exposure was 17.2 years (a range of 2 years, 2 months–30 years). Despite claims that TSW is simply untreated AD, two of our patients had no prior history of AD. Additionally, while there exists controversy surrounding whether patients with disease and TCS exposure limited to their face are equitable to patients with systemic reactions, our patient cohort departed from previous descriptions, exhibiting widespread disease and no patients used TCS improperly, if defined as use of high-potency TCS on the face [8].

However, beyond corticosteroid exposure, it remains difficult to determine which patients are at highest risk of TSW. A series of systematic reviews have found 81.0% and 78.9% of TSW patients to be female, respectively [8,9], which may be skewed by the inclusion of patients who used TCS for cosmetic purposes [9,12]. In our case series, 14 of 16 patients were female, yet none used TCS on the face or for cosmetic benefit.

To date, studies regarding TSW also largely include adults, with just 7.1% and 16.9% of patients being under 18 in a series of systematic reviews and 20.3% in another cross-sectional study [8,9,12]. Amongst adults, younger patients under the age of 35 appear to be overrepresented [12,19]. However, further study is warranted as it is unclear whether this reflects a true biological vulnerability or a reporting bias, as younger adults may be more likely to encounter information about TSW online and self-identify with the condition [4,6,26,27]. Importantly, beyond corticosteroid exposure, there are currently no reliable predictors of who will develop TSW.

Similarly, the natural history of the condition, including its timing and resolution, is variable and unpredictable. Classically, more severe symptoms (described by some as a full-body chemical burn) seem to begin within 14–21 days after sudden discontinuation of high-potency corticosteroids [8]. This was reflected in two patients in our cohort, with one patient even reporting symptoms as early as three days. However, reports where symptoms have occurred within 24–48 h after withdrawal have been described [8]. In other cases, after cessation of topical steroids, a longer “honeymoon” period of 4–6 months can take place before the onset of TSW symptoms [9,28,29]. In one prior report, roughly a year after TSW symptoms appeared, the patient experienced a 2-month remission in symptoms before they reoccurred [28].

Resolution of TSW is also variable, ranging from months to years [11]. One systematic review found that partial clearance began within 0–3 months for 76.7% of patients, though 5.1% did not achieve clearance until 12 months [8]. In our study, at follow-up roughly 27 months past initial evaluation, of eight patients that continued to experience itch, the intensity was described as mild in four, moderate in three, and severe in one; compared with the month prior to survey administration, symptoms were described as unchanged in three, a little better but still present in three, and much better but still present in three of the eight patients. However, given the nature of the follow-up, we were unable to conclusively determine whether such symptoms are attributable to TSW or to other dermatoses instead.

In addition to the uncertainty surrounding its risk factors and resolution, the pathophysiology of TSW has yet to be clearly elucidated. Histological studies have noted epidermal atrophy, spongiosis, and parakeratosis, though these findings are nonspecific to TSW [20]. Current untested theories about its pathology include tachyphylaxis (the decreasing effect of topical corticosteroids after repeated application), glucocorticoid receptor dysregulation, keratinocyte cortisol production dysregulation, rebound vasodilation, or topical corticosteroid induced barrier disruption leading to a cytokine cascade [8].

Extending beyond theoretical models, the first directed mechanistic study of TSW was recently performed. Microbiome analysis, both bacterial and fungal, revealed similarities between TSW and AD [13]. However, further studies involving comparison with other corticosteroid-associated dermatoses with microbiome alterations such as steroid rosacea may help reduce the confounding of TSW with AD [30]. Additional stratification of microbiome profiles by the presence or absence of metallic taste or odor may offer additional insight in future studies; however, microbiome sampling in our cohort was performed at the time of initial presentation rather than at symptom onset, limiting such analyses in the current dataset [13]. Additional comparison of serum metabolomics between patients with TSW and both healthy volunteers and non-TSW AD patients has revealed that TSW patients have significant deficiencies in the sphingolipid and urea cycle amino acid pathways, with significant overabundance of metabolites such as glycerophospholipids and C21-steroid hormones including cortisol [13]. Prior analysis of our cohort’s cytokine profiles revealed that, like AD, patients with TSW had elevated interleukin (IL-) 13, IL-6, and CCL13 compared to controls; however, the levels of these proinflammatory markers were significantly greater in TSW than in patients with AD [13]. Consistent with these inflammatory findings, in our case series, 11 out of 16 patients displayed elevated IgE and 4 out of 11 exhibited elevated eosinophil counts. In addition to these immunologic markers, two patients in our study also experienced new hypersensitivities to foods. Recent allergen testing has shown that, compared with AD patients, those with TSW exhibit higher specific IgE levels to allergens such as dust mites, pet dander, and fungi, and a larger proportion test positive for at least one epitope within aeroallergen categories [31]. However, further study is warranted to better understand if TSW may increase sensitization risk, or conversely, if pre-existing sensitization might contribute to the pathophysiology of TSW.

Additional skin metabolomics has also revealed that amongst TSW patients, vitamin B3 (nicotinate and nicotinamide) metabolism was significantly increased, while tryptophan metabolism, an amino acid from which B3 is derived, was significantly downregulated [13]. Upregulation of nicotinate metabolism in TSW was also corroborated by RNA-seq analysis and appears to be derived from an overexpression of the mitochondrial complex I [13]. NAD+ is known to interact with itch-regulating transient receptor potential (TRP) family ion channels and staining of TRPA1 uncovered increased dermal but not total expression in TSW patients [13]. Application of nicotinic acid to mouse ears yielded a distinct pattern of erythema compared to existing AD murine models, with erythema localized to areas with hair [13]. Additionally, given the absence of erythema in the nasal, palmar, and plantar regions, along with the delayed resolution of TSW, this may indicate a role for stem cells in the pathology of TSW, as they are located at the base of each hair follicle and act as progenitors for replenishing other skin cells [13].

In vitro models of human follicular stem cells (HFSC), keratinocytes, fibroblasts, and Schwann nerve cells revealed that incubation with steroids did not impact B3 metabolism, while subsequent withdrawal did [13]. Altogether, such results indicate that TSW may be caused by overactivation of complex I, increasing NAD+ oxidation through potentially heightened complex I expression or greater NADH availability via tryptophan metabolism [13].

In support of this theory of increased hyperactive complex I and NAD+ oxidation underlying TSW pathology, the known complex I inhibitors berberine and metformin have improved self-report measures of symptoms [13]. Berberine’s mechanism of action may also be derived from inhibition of genes related to TNF-α, AGE-RAGE, and inflammatory signaling pathways in the context of Staphylococcus aureus infection, though further work using in vitro models is needed to further shed light on the potential mechanisms [32]. Wu Mei Wan, a traditional Chinese medicine containing berberine as an active ingredient, has also improved symptoms in TSW patients [33]. Methylene blue, a modulator of mitochondrial function, has also circumstantially improved symptoms in one case report [34]. While traditionally regarded as an enhancer of mitochondrial function, cell culture models have shown it to bypass complex I. Coenzyme Q10, another complex I bypass inducer, may carry theoretical benefit in TSW; however, such mechanisms for both methylene blue and Coenzyme Q10 remain speculative and further study is required [34].

Despite the promise of berberine and methylene blue, effective management strategies for TSW remain unclear, with existing studies involving small sample sizes and often lacking controls and standardized outcome measures. Moreover, in patients with coexisting inflammatory dermatoses such as AD, interpretation of therapeutic outcomes may be partly confounded by underlying dermopathies.

Unsurprisingly, systemic steroids are effective, as these suppress the inflammation upstream of many inflammatory pathways [11]; however, oral steroids notably cause significant rebound flares upon course completion and are not a reasonable treatment option for these patients due to harmful side effects with prolonged use. Thus far, across case series, dupilumab has also shown promise [3,35,36]. Its effectiveness was variable amongst our patients, with four out of six patients trialing the medication noting relief, though two of these patients experienced significant enough side effects to warrant discontinuation. Other case reports and series have highlighted 10% topical tranexamic acid, ruxolitinib, and the systemic immunosuppressants methotrexate and cyclosporine as treatments with potential, though further research is warranted [3]. In our case series, one patient trialed topical ruxolitinib for eight weeks without substantial relief. Another trialed cyclosporine, noting just minimal relief. Furthermore, immunosuppressants such as cyclosporine and methotrexate come with a significant risk of adverse side effects [37].

Another preliminary study of five TSW patients by Wallen-Russell et al. aimed to address TSW symptoms by increasing microbiome diversity via dietary changes and the use of a face and body wash previously shown to increase microbiome diversity, with patients’ self-reported symptom severity decreasing [38]. However, further investigation with a larger sample, controls, and validation of microbiome diversity changes is required to support the efficacy of this intervention.

Other common management strategies employed to address TSW include the discontinuation or gradual weaning of topical steroid use, antibiotics, tetracycline, erythromycin, antihistamines, topical calcineurin inhibitors, neuropathic pain agents, UV therapy, wet or dry dressings, topical emollients, and ice and cool compresses [3,8,9]. A case report of two patients documented resolution following cessation of topical corticosteroids, although recovery took approximately two years, highlighting the need for research into more effective and timely therapeutic options [39].

In addition to the lack of established treatment approaches, the skepticism or limited acceptance of TSW within the medical community poses significant challenges for patients. Some patients describe fear of dismissal after presenting their concerns regarding TSW [19,40], while others avoided physicians due to a sense of “anger” and “hatred,” or expressed distrust of physicians from not being warned of TSW as a possible outcome of topical steroid use [40]. To cope, many seek support amongst others with TSW, while others avoid sharing their perspectives on online forums out of a sense of embarrassment [40]. The International Topical Steroid Awareness Network support group page has grown to 27,000 followers worldwide, speaking to the interest in community building among those self-diagnosed with TSW [41]. Similarly, due to feelings of dismissal from dermatologists, many patients turn to alternative or non-traditional medicine for treatment [12,19,40], as reflected in our study, sometimes at a significant financial or health risk. This mistrust not only delays appropriate care but may also deter patients from participating in research, thereby perpetuating existing gaps in knowledge.

Our study has several limitations, including its small sample size and single-center design, which restrict the generalizability of the findings. Assessment of symptomatology and treatment response relied on patient self-report and given the prolonged course of corticosteroid use and TSW, these data are subject to recall and reporting bias. Many patients in our cohort presented after refractory supportive treatments and at longer durations since steroid cessation (mean 3.9 years; median 2.4 years), which may limit generalizability to TSW cases encountered earlier in clinical practice. Interpretation is further complicated by heterogeneity in corticosteroid exposure. Moreover, the off-label and experimental therapies used by some patients were trialed in small numbers and without standardized protocols or outcome measures, limiting the strength of conclusions regarding their effectiveness.

In summary, this case series contributes to the rapidly growing body of literature on topical steroid withdrawal, highlighting features that were not previously well described, including the unusual metallic odor of affected skin, frequent elevations in IgE and eosinophil counts, and new food hypersensitivities. We also report on the psychosocial burdens and the use of various off-label therapies, which demonstrated variable benefit among patients. While our findings support the recognition of TSW as a distinct clinical entity, the lack of standardized diagnostic criteria, validated outcome measures, and established treatments underscores the need for larger, prospective investigations. Advancing understanding of this condition will require improved recognition, consensus definitions, and systematic evaluation of therapeutic strategies to better guide management and improve patient quality of life.

## Figures and Tables

**Table 1:** Clinical Characteristics and Corticosteroid Exposure History.

	Age	Gender	Race	Prior History of AD	Exposure Time	Time Since Discontinuation
Case 1	27	F	Asian	Yes	2 years, 2 months	1 year, 7 months
Case 2	50	F	White	Yes	26 years	11 years
Case 3	51	F	White	No	20 years	7 years
Case 4	36	F	Asian	Yes	27 years	4 years, 3 months
Case 5	38	F	White	Yes	29–30 years	3 years, 2 months
Case 6	58	M	White	No	5 years, 9 months	3 years, 3 months
Case 7	21	M	White	Yes	16 years	6 months
Case 8	27	F	Asian	Yes	“On/off for many years”	8 months
Case 9	34	F	White	Yes	“On/off for 21 years”	8 years, 6 months
Case 10	40	F	Black	Yes	20 years	4 months
Case 11	46	F	White	Yes	2–3 years	10 years
Case 12	35	F	White	Yes	“20+ years”	More than 6 months
Case 13	33	F	Asian	Yes	12 years, 8 months	1 year, 8 months
Case 14	29	F	White	Yes	16 years	19 months since last oral, 24 months since last topical
Case 15	41	F	White	Yes	30 years (17 consecutive)	7 years, 8 months
Case 16	28	F	White	Yes	23 years	14 months

AD: Atopic Dermatitis.

**Table 2:** Reported symptoms and frequencies in study cohort.

Symptom	Frequency, *n* (%)
**Burning**	16 (100)
**Heat/sun sensitivity**	16 (100)
**Itch**	16 (100)
**Pain**	16 (100)
**Shedding**	16 (100)
**Erythema**	16 (100)
**Dryness**	16 (100)
**Scaling**	16 (100)
**Elephant skin**	15 (94)
**Swelling/edema**	15 (94)
**Thermodysregulation**	14 (88)
**Nerve symptoms (zingers)**	14 (88)
**Oozing**	13 (84)
**Metal smell**	9 (56)
**Hair loss**	9 (56)
**New allergies**	8 (50)
**Swollen lymph nodes**	8 (50)
**Insomnia**	7 (44)
**Joint pain**	7 (44)
**Palpitations**	6 (38)
**Unusual smell**	5 (31)
**Vision changes**	4 (25)
**Papules**	1 (6)
**Pustules**	1 (6)
**Skin tightness**	0
**Telangiectasia**	0

**Table 3:** Follow-up symptom activity and functional impact among respondents.

Domain	Response Category	*n* (%)
Daily itch duration	Less than 6 h/day	3 (38%)
	6–12 h/day	3 (38%)
	12–18 h/day	2 (25%)
Itch intensity	Mild	4 (50%)
	Moderate	3 (38%)
	Severe	1 (13%)
Change since prior month to survey administration	Much better but still present	2 (25%)
	A little better but still present	3 (38%)
	Unchanged	3 (38%)
Impact on sleep	Never or not applicable	4 (50%)
	Rarely affects	
	Delays falling asleep and causes awakenings	3 (38%)
	Occasionally delays falling asleep	1 (13%)
Impact on leisure/social activities	Never or not applicable	3 (38%)
	Rarely affects	2 (25%)
	Occasionally affects	2 (25%)
	Always affects	1 (13%)
Impact on household/errands	Never or not applicable	4 (50%)
	Rarely affects	1 (13%)
	Occasionally affects	2 (25%)
	Always affects	1 (13%)
Impact on work/school	Never or not applicable	5 (63%)
	Rarely affects	0 (0%)
	Occasionally affects	2 (25%)
	Always affects	1 (13%)

## Data Availability

The data presented in this study are available on request from the corresponding author due to privacy restrictions.

## Supplementary Materials

The following supporting information can be downloaded at https://www.mdpi.com/article/10.3390/jcm15010361/s1, File S1. TSW case reports; Table S1: Topical steroid treatment; Table S2: Follow-up survey symptoms and functional impacts; Table S3: Absolute Eosinophil Counts.

## Abbreviations

The following abbreviations are used in this manuscript:

AD	Atopic Dermatitis
DLQI	Dermatology Life Quality Index
HFSC	Human Follicular Stem Cells
TSW	Topical Steroid Withdrawal
