# Supplementary material for: Examining the Unanswered Questions in TSW: A Case Series of 16 Patients and Review of the Literature
Source: J Clin Med. 2026 Jan 3;15(1):361. doi: 10.3390/jcm15010361 (PMC12787187; doi:10.3390/jcm15010361)
Supplement: Supplementary file 1 [file jcm-15-00361-s001.zip › Supplemental File S1. TSW case reports.pdf]

Supplemental File S1. TSW case reports

Case 2

50-year-old female with a past medical history of Atopic Dermatitis (AD) who was initially prescribed topical steroids for treatment of AD. She usually applied steroids to her hands, neck, wrist, and arms. Her total steroid exposure time was intermittent for 26 years with the last 10 years including Protopic (tacrolimus) usage as well. She discontinued steroids 11 years prior to initial evaluation at National Institutes of Health (NIH) and initially experienced symptoms of burning, heat sensitivity, itch, pain, hot flashes, dryness, "elephant skin”, abnormal sensations, erythema, papules, pustules, swelling/edema, dryness, scaling, and metallic smelling skin oozing a milky liquid. She also felt “zingers” (neuropathic pain), lymphadenopathy, and insomnia. She developed new-onset hypersensitivity after eating fish which was not previously present. She also had oral allergies with pistachios and cashews which was new. Her symptoms were most severe between 2012-2013. At the time of initial evaluation, her symptoms included occasional flares on arms and hands and her lab work was significant for an elevated total IgE level of 2255 IU/mL.

Case 3

52-year-old female without any past history of atopic dermatitis. She was prescribed steroids initially due to a suspected contact dermatitis to mascara. She was prescribed both prednisone for the eye reaction and topical steroids for a rash along her jaw. The rashes persisted and she was given clobetasol. She also used desonide on the face, which resolved the face rash briefly, but the rash returned days later. Her total steroid exposure time was about a year and half prior to discontinuation of all steroids 7 years prior to initial NIH evaluation (brief exposure in 2017). Her steroid withdrawal symptoms included red and "angry" skin, and several months later, she developed severe pruritus. Symptoms presented above waistline, with nothing on the legs or feet. She continued to have a dry pruritic rash that disrupts her sleep. She tried dupilumab for a ten-month period from 2019 and 2020 with some improvement but discontinued it due to conjunctivitis. Her primary issue was pruritus and disturbed sleep often causing multiple nighttime awakenings.

Case 4

36-year-old female with a history of childhood-onset AD who used topical corticosteroids as needed with effective symptomatic relief. When she was in nursing school (around age 20-years-old), she developed worsening erythema with dry skin involving her hands, which she thought to be contact dermatitis given frequent handwashing. She applied topical corticosteroids on her off days with some symptomatic improvement but continued to have rashes and pruritus during pregnancy that she treated with topical steroids. Postnatally, her dermatologic symptoms persisted, and she experienced worsening skin changes (redness, dryness and scaling) in the perioral area, along with a burning sensation. She trialed dupilumab for 4 months in 2018 without improvement and discontinued due to conjunctivitis. She became concerned for TSW and stopped all steroid usage in 2019. Her total steroid exposure time was 27 years and her time since ceasing steroids had been over 4 years at initial NIH evaluation. Her immediate withdrawal symptoms included whole body

erythema with swelling and hot flushing, along with night sweats, temperature sensitivity, insomnia, and poor appetite. Her symptoms at the time of NIH evaluation include facial and upper extremity rash (particularly of the hands) with redness, dryness, scaling, and burning sensation. Her laboratory workup was significant for an elevated total IgE level of >10,000 IU/mL in June 2023.

Case 5

38-year-old female with past medical history of childhood AD intermittently treated with topical corticosteroids with good symptomatic relief. Her total steroid exposure time was intermittent for thirty years and she stopped steroid use prior to initial evaluation. Her immediate withdrawal symptoms included severe skin rash with burning, heat sensitivity, pruritus, pain, hot flashes, elephant skin, zingers, skin shedding, erythema, swelling, edema, dryness, oozing, scaling, and hair loss. Upon evaluation she continued to experience a burning sensation, heat sensitivity, erythema, dryness and scaling of her skin, mainly on her arms and neck.

Case 6

59-year-old male with no past medical history of atopic dermatitis. He initially used topical steroid cream on his palm after developing a rash in 2015. The rash resolved with treatment, but he continued to experience recurrent rash with discontinuation of topical steroids which progressed along his palm and forearm. Upon stopping the more potent topical steroid, he developed a full-body rash similar to hives. He was subsequently prescribed clobetasol (high-potency steroid) which he took for two years. His total steroid exposure time was 5 years and 9 months prior to discontinuation, and he did not have any steroid exposure for 3 years by the time of initial NIH evaluation. His immediate withdrawal symptoms in 2019 included full-body rash, oozing, severe pain, and sensation of chemical burn. At evaluation he continued to have intermittent flares of his hands and arms with significant swelling of digits, hypopigmentation, erythema, and severe itch without oozing or pus.

Case 7

21-year-old male with past medical history of childhood AD treated intermittently with steroids and Protopic. In high school he used clobetasol and prednisone for symptom management. His total steroid exposure time was intermittent for 16 years. His time off steroids was about six months at the time of NIH evaluation. He had also recently trialed dupilumab for 6 months with symptom improvement. However, he discontinued use after developing facial vasculitis. He also tried Rinvoq (Upadacitinib) for 5 months, which helped for one month before improvements ceased. His immediate steroid withdrawal symptoms included burning, heat sensitivity of skin, itching, pain, hot flashes, elephant skin, zingers, shedding, erythema, swelling, edema, dryness, oozing, scaling, and metallic taste and smell. At the time of NIH evaluation symptoms included insomnia, rash, pruritus and were managed with cyclosporine without significant relief.

Case 8

27-year-old female with past medical history of AD treated intermittently with topical steroids since childhood. She started more regular use of hydrocortisone for hand atopic dermatitis roughly two years prior to initial evaluation along with Protopic. After about a year of regular use, she noticed a worsening and spreading rash. She subsequently stopped all steroid use 8 months prior to initial evaluation. Her immediate symptoms post-steroid discontinuation included a spreading rash all over her body. Additionally, she experienced burning, itching, pain, hot flashes, elephant skin, shedding, erythema, swelling, edema, dryness, oozing (somewhat), scaling, metallic taste and smell, hair loss, new allergies/sensitivities to sunscreens, shrimp, and tomatoes. She also reports insomnia. At the time of evaluation her rash had somewhat improved and was no longer spreading, but she continued to have flares in her face, upper extremities, and hands.

Case 9

34-year-old female with past medical history of childhood AD treated intermittently with topical steroids. When she was 23 years old, she had more severe flares requiring higher potency steroids along with Protopic for management. She continued intermittent usage for 3 years but noticed a spreading rash after discontinuation. Her total steroid exposure time was intermittent for 21 years. She subsequently stopped because of suspicion for TSW. Her time from steroid exposure was 8 years at initial NIH evaluation. Her immediate withdrawal symptoms upon steroid discontinuation included a full-body rash. She also experienced burning, some heat sensitivity, itching, pain, hot flashes, elephant skin, shedding, erythema, swelling, edema, dryness, oozing (somewhat), scaling, metallic taste and smell, hair loss, and insomnia. She had experienced overall improvement at the time of initial NIH evaluation and had been managing her symptoms with Dupilumab for the past six months with some relief.

Case 10

40-year-old female with past medical history of childhood AD treated intermittently with topical steroids. In her twenties, she developed a more diffuse rash all over her body, which was treated with clobetasol. She subsequently had a more severe rash upon discontinuation of high-potency steroids. Her total steroid exposure time was twenty years and her time since last steroid exposure was four months by the time of initial NIH evaluation. Her symptoms immediately upon discontinuation included rash along with burning, heat sensitivity, itching, pain, cold flashes, elephant skin, shedding, erythema, swelling, edema, dryness, oozing, scaling, and a strange smell of skin. She also experienced zingers, lymphadenopathy, insomnia, and hair loss.

Case 11

46-year-old female with past medical history of childhood AD, previously well-controlled with non-steroidal topical ointment developed AD-like lesions on her hands after applying steroids (desonide) to her son who also suffers from AD. In the next year, she had more AD flares and increased steroid usage, however, steroid discontinuation resulted in worsening of her rash. Her last steroid exposure was ten years ago, and her immediate withdrawal symptoms included a full-body severe rash that made her feel like a “burn victim.” She had extreme pruritus, pain, oozing, and recalls a strong odor. As flares subsided, the rash would become dry, crusty, with elephant

skin and extensive skin shedding. She tried many therapies during this period including IV chelation therapies, infrared sauna therapy, acupuncture, and natural supplements without much relief. She also underwent a "stem cell" treatment in a holistic facility with umbilical stem cells and afterwards noted significant improvement within a week. She used Zoryve for 4 weeks as part of a study to treat an ankle rash which initially provided relief before it was discontinued. Her clinical symptoms at the time of initial NIH evaluation included severe rash on her hands bilaterally and on her right ankle for which she was doing biofeedback and neurofeedback trials along with homeopathic supplements.

Case 12

35-year-old female with past medical history of childhood AD treated with intermittent topical steroids. She had worsening rash that spread during her early 20s treated with high-potency steroids such as betamethasone, fluocinonide, and clobetasol. She had a total steroid exposure time which involved intermittent use for over 20 years, and her time since steroid exposure was six months at the time of initial NIH evaluation. Her immediate withdrawal symptoms included severe rash of arms and chest along with burning, heat sensitivity, itching, pain, hot flashes, elephant skin, shedding, erythema, swelling, edema, dryness, oozing, scaling, putrid smell, zingers, lymphadenopathy, and fuzzy vision.

Case 13

33-year-old female with past medical history of AD since childhood treated with intermittent topical steroids. Her total steroid exposure time included intermittent use for 12 years and no exposure to steroids for 1 year and 8 months at the time of NIH evaluation. Her immediate withdrawal symptoms included an erythematous, warm, and pruritic rash on her extremities and face. She had oozing areas of skin with eventual healing, dry pruritic skin, and increased skin shedding. Her genetic testing proved unrevealing.

Case 14

29-year-old female with past medical history of childhood AD treated intermittently with high-potency steroids such as clobetasol. Her total steroid exposure duration included intermittent use over 16 years and her time since last steroid exposure was 19 months at the time of NIH evaluation. Her immediate withdrawal symptoms included diffuse severe rash along with burning, heat sensitivity, pruritus, pain, cold flashes, elephant skin, shedding, erythema, swelling, edema, dryness, oozing, scaling, metallic smell, zingers, lymphadenopathy, insomnia, and hair loss. Given a lack of effective therapies, she had also trialed an eight-week course of topical ruxolitinib cream without much help. At the time of NIH evaluation, she also trialed dupilumab with symptom relief.

Case 15

41-year-old female with a past medical history of AD treated intermittently with topical steroids. She also received steroid injections in her scalp during childhood as treatment for alopecia. Her total steroid exposure time at NIH evaluation involved intermittent steroid use for 30 years, and her time since last steroid exposure was 7 years. Her immediate post-steroid

discontinuation symptoms included a severe full-body rash. She also experienced burning, heat sensitivity, pruritus, pain, hot flashes, elephant skin, skin shedding, erythema, swelling, edema, dryness, oozing, scaling, abnormal smell, zingers, lymphadenopathy, insomnia, and hair loss. She trialed dupilumab for 6 months and noticed some improvement in the first 6 weeks of treatment before symptoms recurred. She had a past history of eosinophilia with a highest absolute eosinophil count of 1650. She did not have elevated serum IgE levels. At the time of her initial NIH evaluation, she continued to have intermittent flares on arms, legs, neck, and chest and took ibuprofen for pain which provided relief. Her genetic evaluation was clinically insignificant.

Case 16

28-year-old female with past medical history of AD treated with intermittent topical steroids. Her total steroid exposure time included intermittent use over 23 years, and she had not had any steroid exposure for 14 months at the time of NIH evaluation. Her immediate withdrawal symptoms included severe erythematous rash on her face and arms, along with oozing, crusting, flaking, and insomnia. On initial evaluation, she had a mild rash involving her thigh, arms, breast, neck, and sometimes abdomen. She found some relief with a nighttime dose of Naltrexone. Her genetic testing was also unrevealing for reportable conditions.
